# Align and Fuse: A Transformer-Based Framework for EEG-Augmented Visual Recognition

**DOI:** 10.3390/brainsci16070723

**Published:** 2026-07-07

**Authors:** Chao Zhang, Youpeng Ma, Mengting Li, Xiangping Gao, Xiaopei Wu

**Affiliations:** School of Computer Science and Technology, Anhui University, Hefei 230601, China

**Keywords:** electroencephalography, visual recognition, multimodal fusion, contrastive learning, Transformer, brain-computer interface, deep learning

## Abstract

**Highlights:**

**What are the main findings?**

- Align and Fuse provides a two-stage neuro-visual framework that first aligns EEG and image representations with hardness-aware contrastive learning and then fuses them through a co-attention Transformer for EEG-augmented inference.
- Under a temporally separated EEG-ImageNet control protocol, the framework achieves 91.12% Top-1 accuracy, while the original stratified random split yields 92.56%; on EEGCVPR, it achieves 95.82%, with 90.92% average cross-subject accuracy on unseen subjects.

**What are the implications of the main findings?**

- Human EEG signals can provide complementary stimulus-related information that may help computational vision models resolve ambiguity between coarse- and fine-grained visual categories when EEG is available at inference time.
- Hardness-aware neuro-visual alignment may support more robust human-in-the-loop EEG-augmented visual recognition and improve the generalizability of BCI-related recognition systems.

**Abstract:**

**Background**: Integrating human neural signals with computational vision systems offers a promising route toward more robust visual recognition, yet supporting mixed-granularity recognition, where both coarse- and fine-grained categories must be distinguished within a unified system, remains challenging due to the heterogeneous feature spaces of electroencephalography (EEG) and visual data. **Methods**: We propose “Align and Fuse,” a two-stage Transformer-based framework. Stage 1 constructs a shared semantic space using a hardness-aware multimodal supervised contrastive loss with Hard Negative Weighting to explicitly target confusable class pairs. Stage 2 employs a multimodal Transformer with co-attention to fuse the aligned features for classification. **Results**: On the 80-class EEG-ImageNet benchmark, our framework achieved 91.12% Top-1 accuracy under a temporally separated control protocol, improving over the corresponding vision-only (89.08%) and Standard Transformer (89.95%) baselines. Under the original stratified random split, it achieved 92.56% Top-1 accuracy; on the 40-class EEGCVPR dataset, accuracy reaches 95.82%. Cross-subject experiments yield 90.92% average Top-1 accuracy on four unseen subjects, and Grad-CAM analysis suggests that aligned EEG signals shift the model’s attention toward semantically relevant regions. **Conclusions**: Coupling hardness-aware alignment with decoupled multimodal fusion supports EEG-augmented recognition by leveraging complementary stimulus-related information under the evaluated protocols. Because EEG features are required at inference time, the framework is positioned as a human-in-the-loop EEG-augmented recognition system rather than a standalone vision model.

## 1. Introduction

The rapid advancements in deep learning have empowered computer vision systems with remarkable capabilities, often rivaling human performance in standard object recognition tasks [1,2]. However, these artificial systems still face difficulty in robust mixed-granularity recognition, where a unified model must distinguish both broadly different (coarse-grained) and highly similar (fine-grained) categories. Standard models often struggle with the subtle, category-defining details required for fine-grained distinctions; moreover, addressing the mixed-granularity challenge demands even greater flexibility and robustness. This gap raises a practical question: can non-invasive neural signals provide complementary information that helps visual models resolve such ambiguity? Brain-Computer Interfaces (BCIs), particularly those based on non-invasive electroencephalography (EEG), offer a promising pathway by capturing the neural dynamics of visual perception in real-time [3]. As highlighted in a recent review [4], combining neural decoding with multimodal learning is becoming an important direction for developing neuro-inspired machine perception systems.

The endeavor to develop EEG-augmented visual recognition systems is built upon a strong foundation of progressively sophisticated methods. Pioneering studies, such as Spampinato et al.’s work, reported that EEG signals can support visual classification under controlled EEG-image protocols [5]. Cudlenco et al. [6] demonstrated that fusing EEG with visual features can improve recognition accuracy, suggesting that brain signals provide useful complementary information. However, these early approaches were constrained by the disparate feature spaces of the two modalities. A significant breakthrough came with the adoption of the “align before fuse” paradigm, originally popularized in vision-language learning [7,8]. By adapting this strategy, researchers began using contrastive learning to map EEG and image representations into a shared semantic space. Recent works, including those leveraging supervised contrastive learning [9], have successfully established this alignment as a foundational step. Nevertheless, three limitations remain insufficiently addressed for mixed-granularity EEG-augmented visual recognition. First, existing EEG-image alignment methods often optimize global cross-modal matching but do not explicitly emphasize confusable fine-grained categories. Second, although supervised contrastive learning can use class labels to improve semantic clustering, standard formulations do not explicitly reweight hard negatives that arise from visually similar categories in mixed-granularity EEG-image recognition. Third, if multimodal fusion is trained without preserving the aligned geometry, the benefits of alignment may be weakened by unstable cross-modal optimization. These limitations motivate an alignment strategy that is hardness-aware and a fusion strategy that operates on a stable, pre-aligned representation space.

**Figure 1:**
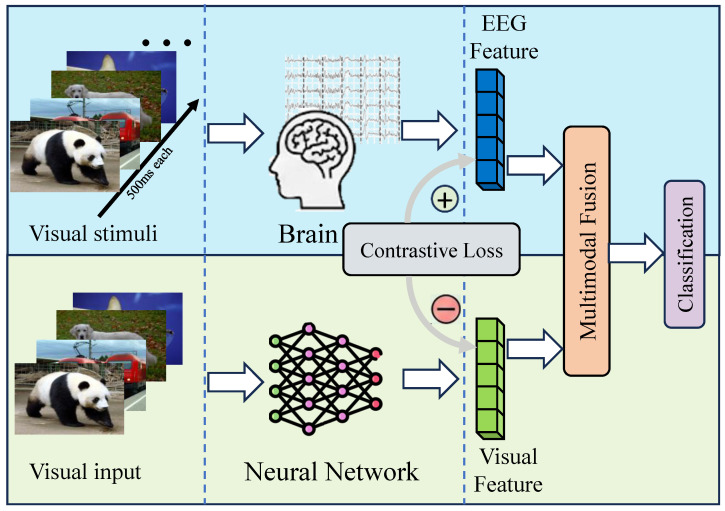
The “Align and Fuse” paradigm for EEG-augmented visual recognition. By aligning and fusing EEG and image streams, the framework uses EEG-derived stimulus-related cues to complement visual representations, yielding recognition gains under the evaluated protocols.

This paper contends that the next leap in performance requires tackling both the fine-grained alignment and deep fusion challenges simultaneously. To this end, we introduce “Align and Fuse,” a novel two-stage framework that separates shared-geometry construction from multimodal decision fusion. Figure 1 depicts the overarching philosophy of our approach: by aligning and fusing EEG and image representations, the framework incorporates EEG-derived complementary stimulus-related cues into visual recognition. In Stage 1, we propose a Multimodal Supervised Contrastive Learning objective. Rather than relying on a standard contrastive loss, we employ a composite loss designed to jointly optimize for inter-modality alignment and intra-modality clustering. Crucially, this objective incorporates a novel Hard Negative Weighting mechanism [10]. This mechanism assigns greater emphasis to confusable class pairs, which is important for learning decision boundaries in fine-grained categories. During this stage, the EEGConformer and the modality-specific projection layers are trained for contrastive alignment, whereas the ResNet-101 visual backbone is kept frozen to preserve its pre-trained visual representations. In Stage 2, both modality encoders are frozen to preserve the aligned representation geometry. A sophisticated multimodal Transformer, featuring co-attention, is then trained exclusively to model the complex interactions between these fixed features for final classification. This “learn, freeze, and fuse” strategy allows the framework to first construct a discriminative shared semantic space and then perform fusion without destabilizing the fine-grained alignments learned in Stage 1.

To evaluate our approach, we employ two public datasets: the classic 40-class EEGCVPR benchmark [5] and the recent large-scale EEG-ImageNet [11]. We specifically leverage EEG-ImageNet’s unique design, which features a challenging mix of 40 coarse-grained and 40 fine-grained categories. We use the full 80-class task as a challenging mixed-granularity evaluation setting because it requires a model to simultaneously manage both broad semantic separation and subtle detail discrimination. Therefore, we focus our evaluation on this demanding setting to assess the robustness and flexibility of our framework under the evaluated EEG-image protocols.

**Novelties and Contributions.** The main novelties and contributions of this study are summarised as follows:

- We formulate mixed-granularity EEG-augmented visual recognition as a neuro-visual learning problem in which coarse-grained semantic separation and fine-grained category discrimination must be handled within a unified framework.
- We propose a hardness-aware multimodal supervised contrastive alignment strategy. By incorporating Hard Negative Weighting, the shared EEG-image semantic space is explicitly shaped around confusable class pairs, distinguishing our method from standard contrastive alignment that treats negative samples more uniformly.
- We introduce a decoupled “learn, freeze, and fuse” strategy with a co-attention Transformer. This design preserves the aligned representation geometry learned in Stage 1 while enabling the fusion module to model cross-modal interactions for classification.
- We complement the proposed framework with controlled evaluations across EEG-ImageNet and EEGCVPR, including same-protocol baselines, ablation studies, held-out-subject testing, leakage-control analyses, paired statistical tests, HNW sensitivity analysis, and encoder-robustness analyses to assess EEG contributions under stricter experimental conditions.

## 2. Related Work

### 2.1. Complementarity Between Brain and Machine Vision

Human vision differs from current machine vision systems in its ability to seamlessly integrate low-level sensory input with high-level semantic perception, even under conditions of ambiguity or fine-grained similarity [12]. The primate visual system reflexively processes visual stimuli through a hierarchical cortical pathway, from early feature extraction in low-level visual areas (e.g., V1) to object-level categorization in higher-level ventral regions such as the inferior temporal cortex (IT) [13]. In contrast, conventional deep learning models—despite structural similarities to these cortical hierarchies [14]—tend to operate primarily on pixel-level patterns and are highly susceptible to brittle failures under slight perturbations [12]. More importantly, a large-scale study by Rajalingham and DiCarlo [15] revealed that even when Deep Convolutional Neural Networks (DCNNs) match primate performance on average, they diverge significantly at the level of high-resolution, image-level behavioral consistency. In other words, models often fail on specific images that are trivial for biological systems. This discrepancy implies that the human brain relies on distinct, robust visual processing mechanisms not yet captured by standard DCNNs. Consequently, they often exhibit drastically different error patterns compared to humans and lack the innate ability to make context-aware distinctions between visually similar instances.

Electroencephalography (EEG) offers a complementary perspective: as a direct measurement of cortical activity, EEG encodes the brain’s dynamic response to visual input in both time and space. It may contain perceptual, attentional, and task-related information associated with visual processing, even when no explicit task is given [5]. Recent studies have demonstrated that EEG signals contain category-specific information that can be decoded at recognition-level accuracy using deep neural architectures [16,17,18]. Crucially, evidence for the synergy between these neural signals and visual features has been provided by pioneering works such as Cudlenco et al. [6], who demonstrated that combining EEG with image embeddings significantly improves recognition accuracy. Notably, they found that improvements stemmed largely from higher cognitive areas (frontal and parietal lobes) rather than the visual cortex, suggesting that EEG may provide complementary information beyond low-level visual representations. Generative works such as Brain2Image [19] further validate the richness of these signals by demonstrating the possibility of reconstructing visual content directly from neural activity. However, despite these promising indicators, the majority of research remains focused on decoding EEG alone, often overlooking the systematic exploitation of its synergy with image-based features. To fully leverage this complementarity, a fundamental prerequisite is establishing a robust correspondence between these disparate modalities—a challenge that has driven the evolution of representation alignment techniques.

### 2.2. Representation Alignment Between EEG and Visual Modalities

To enable effective cross-modal fusion, mainstream approaches typically employ contrastive learning to map representations into a shared embedding space. Early works, such as Palazzo et al. [20], utilized Siamese networks to explicitly minimize the distance between corresponding EEG-image pairs. This paradigm was solidified by foundational works like Contrastive Predictive Coding (CPC) [21] and the success of CLIP [8] in vision-language learning. Recent adaptations, including EEG-CLIP [22] and NICE-EEG [23], have successfully transferred this framework to the neuro-visual domain. While these methods effectively learn a joint space for cross-modal retrieval (e.g., matching EEG to images) or generative tasks, they typically lack a downstream fusion mechanism designed for discriminative decision-making. This limitation prevents them from fully exploiting the complementary nature of EEG for enhancing visual recognition accuracy. Beyond point-to-point alignment, researchers have also explored capturing deeper structural correlations, such as disentangling common and private features [24] or preserving the relational topology of samples [25], to better transfer EEG-derived stimulus-related information to vision.

Recent progress has increasingly leveraged label information through Supervised Contrastive Learning (SCL). For instance, Quan et al. [9] proposed a composite SCL objective optimizing for both inter- and intra-modality consistency. However, a critical bottleneck remains in the handling of negative samples. Theoretical studies [10] argue that standard uniform sampling is suboptimal, as it is dominated by “easy” negatives that provide vanishing gradients. For mixed-granularity tasks, this uniform penalty is insufficient, as the model lacks the incentive to distinguish highly similar but distinct classes (e.g., *Husky* vs. *Border Collie*). Existing multimodal SCL frameworks often overlook this insight, treating all negatives equally. In this respect, our method differs from both CLIP-style alignment and standard SCL formulations. CLIP-style objectives mainly establish global cross-modal correspondence between paired samples, whereas standard SCL uses class labels to improve clustering but still applies a uniform denominator over negative samples. Our Hard Negative Weighting instead explicitly reweights negative pairs according to their feature-space similarity, thereby increasing the contribution of confusable categories during EEG-image alignment.

To address this limitation, our work enhances the supervised contrastive paradigm by introducing a Hard Negative Weighting mechanism. By dynamically amplifying the penalty for semantically confusable samples, we force the model to learn more refined decision boundaries for fine-grained distinctions. Coupling this hardness-aware alignment with a Transformer-based fusion architecture allows us to construct a robust EEG-augmented recognition framework. Yet, establishing a shared semantic space is only the prerequisite; the ultimate performance hinges on how these aligned features are subsequently integrated to model complex inter-modal dependencies.

### 2.3. Fusion Strategies for EEG-Augmented Visual Recognition

The fusion of information from different modalities has long been a central theme in advancing AI systems toward more context-aware multimodal understanding [26]. A significant advancement in this area has been the introduction of cross-attention mechanisms within Transformer architectures [27], which have proven highly effective in modeling inter-modal dependencies for tasks such as vision-language understanding [28] and, more pertinently, audio-visual tasks including emotion recognition. For instance, recent works like Foal-Net [29] have demonstrated that aligning audio and visual features through contrastive learning before fusing them via cross-attention achieves state-of-the-art performance. Concurrently, researchers have explored leveraging additional physiological signals, such as combining EEG with eye movements [30], further highlighting the potential of integrating neuro-physiological data. Building on these diverse advancements, our work focuses specifically on the fusion of EEG and image data.

Early studies, such as Cudlenco et al. [6], provided compelling evidence for neuro-visual complementarity, showing that simple feature concatenation could substantially boost classification accuracy. However, while these naive strategies (e.g., concatenation or element-wise addition) validated the core concept, their architectural simplicity limited effectiveness. Similarly, Mishra et al. [31] transformed EEG signals into 2D grayscale images to leverage pre-trained CNNs, subsequently stacking extracted features with visual data. Such methods implicitly assume the compatibility of disparate feature spaces, overlooking the complex, non-linear relationships between neural activity and visual inputs.

These limitations have motivated the development of advanced fusion methods that explicitly model inter-modal relationships. Transformers have proven particularly effective due to their ability to capture long-range dependencies. In the EEG domain, Song et al. [18] introduced the EEG Conformer, demonstrating that combining convolution for local features with self-attention for global context yields state-of-the-art decoding performance. While this validates the Transformer’s robustness for neural data, applying such sophisticated architectures to the EEG-Vision task is often hindered by the lack of prior feature alignment. Most existing methods either perform alignment without deep fusion or apply complex fusion on unaligned features, failing to fully exploit the synergy between the two steps.

In this work, we address this challenge by coupling a contrastive alignment module with a Transformer-based fusion architecture. Following the alignment stage—where EEG and image features are projected into a shared semantic space using supervised contrastive learning with hard negative weighting—we freeze the encoders and introduce a cross-attention-based Transformer fusion module to enable fine-grained, context-aware interactions between the modalities. This design allows EEG to guide visual recognition, especially in scenarios involving ambiguous or visually similar instances, ultimately providing a more robust and human-in-the-loop EEG-augmented recognition system.

## 3. Materials and Methods

**Figure 2:**
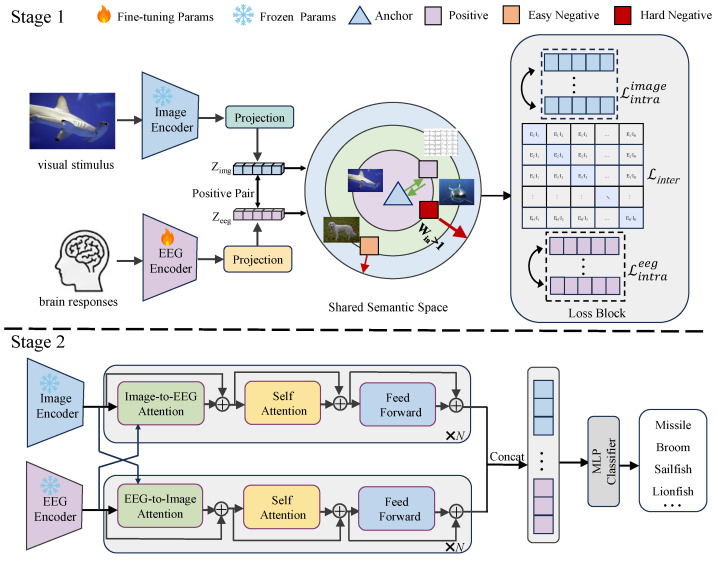
Overview of the proposed “Align and Fuse” framework. Stage 1 aligns EEG and image representations via contrastive learning, explicitly targeting hard negatives (centre, where green/red arrows denote attraction/repulsion) to refine the shared semantic space. Stage 2 fuses these frozen features using a multimodal Transformer for classification.

In this section, we present our novel framework, “Align and Fuse.” Adopting the successful paradigm from vision-language learning [7], our framework is designed to enhance visual recognition by integrating complementary information from EEG signals. The framework operates in two distinct stages: Multimodal Supervised Contrastive Learning, which aims to learn powerful and well-aligned feature representations by training the EEG encoder and modality-specific projection layers while keeping the pre-trained visual backbone frozen; and Transformer Fusion for Classification, which focuses on fusing these learned representations for the downstream task. Crucially, we adopt a decoupled training strategy by keeping the encoders frozen during this stage. This isolates the feature alignment phase from the reasoning phase, ensuring that the fusion module learns to integrate stable, high-quality features without disrupting the established semantic space. This phased approach, as illustrated in Figure 2, allows us to first build a high-quality representation space and then learn the complex interactions within it.

### 3.1. Use of Generative Artificial Intelligence

Generative AI tools, including ChatGPT and Codex (OpenAI), were used only to assist with language polishing, translation, formatting checks, and preparation of submission-related text. These tools were not used to generate original research data, perform experiments, conduct statistical analyses, train models, or draw scientific conclusions. All AI-assisted content was reviewed, revised, and approved by the authors.

### 3.2. Multimodal Supervised Contrastive Learning

The primary objective of Stage 1 is to learn powerful, aligned representations from the raw EEG and visual inputs. To achieve this, our framework employs two distinct encoders: a pre-trained ResNet-101 for the visual modality and an EEGConformer architecture for the neural signals. Following a highly effective transfer learning paradigm that aims to preserve robust, general-purpose knowledge, the backbone of the ResNet-101 encoder remains “frozen” throughout training, with only the projection layers trained. In contrast, the EEGConformer, being a specialized architecture for this domain, is trained from scratch to learn discriminative features directly from our EEG data. Together, these encoders process the input data $x_{img}$ and $x_{eeg}$ to produce high-dimensional feature vectors.

To map heterogeneous EEG and image representations into a shared embedding space, each encoder output is projected through a learnable linear transformation and normalized:

(1) $$\begin{array}{cc} z_{eeg} & =\mathrm{Norm}\;\left(W_{eeg}f_{eeg}\left(x_{eeg}\right)\right),\\ z_{img} & =\mathrm{Norm}\;\left(W_{img}f_{img}\left(x_{img}\right)\right) \end{array}$$

where $W_{eeg}$ and $W_{img}$ are modality-specific projection matrices, and Norm(·) denotes $L_{2}$ normalization to ensure that all embeddings lie on a unit hypersphere. These projected vectors $z_{eeg}$ and $z_{img}$ form the basis for subsequent contrastive learning.

The training of our encoders in Stage 1 is guided by a novel Multimodal supervised contrastive loss, $L_{total}$, which is a weighted sum of two main components:

(2) $$L_{total}=L_{inter}+\beta_{eeg}\cdot L_{intra}^{\left(eeg\right)}+\beta_{img}\cdot L_{intra}^{\left(img\right)}$$

where $\beta_{eeg}$ and $\beta_{img}$ are balancing hyperparameters. Both loss components incorporate our proposed Hard Negative Weighting mechanism. The Inter-modality Loss ($L_{inter}$) operates across modalities to ensure EEG and image features of the same class are aligned. The Intra-modality Losses ($L_{intra}$) operate within each modality to maintain a strong class-discriminative structure. Our key innovation is the introduction of a Hard Negative Weighting mechanism into these loss calculations. This mechanism dynamically amplifies the penalty for semantically confusable negative samples by assigning them a weight $w_{ia}>1$, forcing the model to learn more refined decision boundaries for fine-grained distinctions.

**Inter-modality Loss ($L_{\mathrm{inter}}$).** This loss is the cornerstone of our alignment stage, designed to map EEG and image representations into a shared semantic space. The core principle is contrastive: for an anchor sample from one modality, we pull its positive counterparts from the other modality closer, while pushing away all negative samples.

To formalize this, consider a single EEG embedding $z_{\mathrm{eeg},i}$ as an anchor. The directional loss for aligning this EEG sample to the visual modality is defined as:

(3) $$\begin{array}{cc} L_{\mathrm{inter}}^{\left(\mathrm{eeg}\to \mathrm{img},i\right)}= & -\frac{1}{\left|P(i)\right|}\sum_{p\in P(i)}[\left(z_{\mathrm{eeg},i}\cdot z_{\mathrm{img},p}/\tau \right)\\ \; & -\log\;\left(\sum_{a\in A(i)}w_{ia}\exp\left(z_{\mathrm{eeg},i}\cdot z_{\mathrm{img},a}/\tau \right)\right)] \end{array}$$

where P(i) is the set of positive image embeddings in the batch sharing the same class label as anchor *i*, A(i) is the set of all image embeddings in the batch, and $w_{ia}$ is our proposed hard negative weight.

Crucially, for a robust and stable alignment, this process must be symmetrical. Therefore, to ensure bidirectional consistency between modalities, we define the total inter-modality loss as the average of the two directional losses:

(4) $$L_{\mathrm{inter}}=\frac{1}{2N}\sum_{i=1}^{N}\left(L_{\mathrm{inter}}^{\left(\mathrm{eeg}\to \mathrm{img},i\right)}+L_{\mathrm{inter}}^{\left(\mathrm{img}\to \mathrm{eeg},i\right)}\right)$$

The first term aligns EEG-to-Image embeddings as defined in Equation (3), while the second term enforces the reverse Image-to-EEG consistency by taking an image embedding as the anchor and contrasting it against all EEG embeddings. This symmetric design, inspired by seminal multimodal contrastive frameworks such as CLIP [8], ensures that both encoders are equally incentivized to converge to a truly shared and well-aligned semantic space.

**Intra-modality Loss ($L_{\mathrm{intra}}$).** The intra-modality loss enforces class separation within each modality. Let m∈{eeg,img} denote the modality and B represent the set of indices in the current batch. The loss for modality *m* is defined as:

(5) $$\begin{array}{cc} L_{\mathrm{intra}}^{\left(m\right)}= & -\sum_{i\in B}\frac{1}{\left|P(i)\right|}\sum_{p\in P(i)}[\left(z_{m,i}\cdot z_{m,p}/\tau \right)\\ \; & -\log\;\left(\sum_{a\in A(i)}w_{ia}\exp\left(z_{m,i}\cdot z_{m,a}/\tau \right)\right)] \end{array}$$

**Hard Negative Weighting.** Our key innovation is the introduction of a dynamic weight $w_{ia}$ for each anchor-sample pair (i,a) in the denominator of the loss functions. This weight amplifies the penalty for hard negatives:

(6) $$w_{ia}=\left\{\begin{array}{cc} 1+\lambda_{h}\sigma \left(k\left(z_{i}\cdot z_{a}\right)\right), & \mathrm{if}\;y_{i}\neq y_{a},\\ 1, & \mathrm{otherwise}. \end{array}\right.$$

where $z_{i}$ and $z_{a}$ denote the normalized feature embeddings of the current anchor sample and a corresponding negative sample, respectively. Since these vectors are $L_{2}$-normalized, their dot product $z_{i}\cdot z_{a}$ is equivalent to the cosine similarity. σ(·) is the Sigmoid function, and *k*, $\lambda_{h}$ are hyperparameters controlling the weighting intensity. The intuition behind this mechanism is visually depicted in the central “Shared Semantic Space” module of Figure 2. Unlike standard contrastive learning which treats all negatives uniformly, our formulation explicitly targets “hard negatives”—samples that are semantically close to the anchor yet distinct. Specifically, the proposed weight $w_{ia}$ is inserted into the denominator of both inter- and intra-modality contrastive losses, so hard negatives contribute more strongly to the normalization term than easy negatives. By dynamically assigning these confusable samples a penalty weight $w_{ia}>1$, we generate a stronger repulsive force during optimization. This compels the model to allocate more capacity to distinguishing ambiguous class pairs, thereby learning a highly discriminative embedding space essential for fine-grained recognition.

### 3.3. Transformer-Based Multimodal Fusion

While Stage 1 establishes instance-level semantic correspondence between EEG and visual representations, such alignment alone is insufficient for discriminative decision-making. The geometric proximity learned via contrastive loss does not explicitly capture the high-order inter-modal dependencies required to resolve semantic ambiguities. To address this, the centerpiece of our fusion stage is a multimodal Transformer, $F_{fusion}$, architected as a dual-stream network. This approach, inspired by successful vision-language models like LXMERT [28], processes each modality’s features in a parallel stream while enabling deep, layer-by-layer interaction for contextual reasoning.

The fusion module takes the aligned EEG feature sequences, $H_{eeg}$, and image feature sequences, $H_{img}$, as two separate inputs to their respective streams. The module consists of *L* stacked blocks. Each block is composed of a co-attention layer, a self-attention layer, and a position-wise feed-forward network (FFN), with residual connections and layer normalization applied around each sub-layer.

**Cross-Modal Interaction via Co-Attention.** To explicitly model the complex interactions between modalities, we first introduce a Co-Attention Layer. This layer is crucial for modeling inter-modal dependencies, as it allows features from one modality to attend to the other, enabling cross-modal semantic interaction. Given EEG features $H_{eeg}\in R^{N_{e}\times d}$ and image features $H_{img}\in R^{N_{i}\times d}$, the co-attention mechanism is defined as:

(7) $$\begin{array}{c} H_{eeg}^{\prime }=\mathrm{LN}\left(\mathrm{MH}\left(Q_{eeg},K_{img},V_{img}\right)+H_{eeg}\right)\\ \mathrm{MH}\left(Q,K,V\right)=\mathrm{Concat}\left({\mathrm{head}}_{1},\ldots ,{\mathrm{head}}_{H}\right)W^{O} \end{array}$$

where each attention head is computed as:

(8) $${\mathrm{head}}_{h}=\mathrm{Softmax}\;\left(\frac{Q_{eeg}{\left(K_{img}\right)}^{⊤}}{\sqrt{d_{h}}}\right)V_{img}$$

The query, key, and value projections are given by:

(9) $$\begin{array}{cc} Q_{eeg} & =H_{eeg}W_{h}^{Q}+b_{h}^{Q},\\ K_{img} & =H_{img}W_{h}^{K}+b_{h}^{K},\\ V_{img} & =H_{img}W_{h}^{V}+b_{h}^{V} \end{array}$$

Here, $H_{eeg}$ and $H_{img}$ denote the feature embeddings from the EEG stream and image stream, respectively. LN denotes layer normalization, MH represents multi-head attention, *H* is the number of attention heads, and $W^{O}$ denotes the output projection matrix. While the formula above explicitly shows the EEG → Image attention, in practice we implement a bidirectional co-attention, where $A_{eeg\to img}$ allows EEG features to attend to visual features, capturing which image regions or semantics are most relevant to the given neural pattern. Conversely, $A_{img\to eeg}$ enables the image stream to attend to EEG cues.

**Intra-Modal Refinement via Self-Attention.** Following the cross-modal alignment, a Self-Attention Layer is applied independently to each stream to refine the internal structure of each modality. Unlike standard implementations that might share weights, we explicitly handle the dual-stream inputs by using distinct, modality-specific learnable projection matrices. This separation is critical: it ensures that the EEG stream focuses on modeling temporal and channel-wise dependencies, while the image stream captures spatial structural relationships. Consequently, this step digests the information gained from the co-attention layer and reorganizes it to maintain the semantic coherence of each specific modality without interference.

**Position-wise Feed-Forward Network (FFN).** Finally, the refined features pass through a Position-wise Feed-Forward Network (FFN) to enhance representational abstraction through non-linear transformations. We employ a two-layer perceptron architecture with non-linear activation functions, maintaining the strategy of unique weights for each modality. This stage progressively refines the reasoning ability of the network, transforming the hidden states into stable and discriminative multimodal representations, $H_{eeg}$ and $H_{img}$, which are effectively prepared for the final classification fusion.

After passing through all *L* Transformer blocks, the output embeddings corresponding to the globally representative ‘[CLS]’ tokens from each stream, $h_{eeg\_cls}$ and $h_{img\_cls}$, are concatenated to form the final, comprehensive fused feature vector, $f_{\mathrm{fused}}$. This vector encapsulates the deeply integrated, cross-modally informed representations learned by the fusion module.

This fused representation is then fed into a final classification head, which is typically a multi-layer perceptron (MLP), to produce the probability distribution over the target classes. Formally, given the fused representation $f_{\mathrm{fused}}$, the predicted probability vector $\hat{y}$ is computed as:

(10) $$\hat{y}=\mathrm{Softmax}\left(\mathrm{MLP}\left(f_{\mathrm{fused}}\right)\right)$$

where MLP(·) represents the learnable parameters of the classification head. The final training objective in Stage 2 is to minimize the standard cross-entropy loss between the predicted distribution and the ground-truth labels:

(11) $$L_{cls}=-\sum_{k=1}^{K}y_{k}\log\left({\hat{y}}_{k}\right)$$

where *K* is the number of categories. Here, $y_{k}$ and ${\hat{y}}_{k}$ denote the *k*-th elements of the one-hot encoded ground-truth vector y and the predicted probability vector $\hat{y}$, respectively. During this stage, only the parameters of the multimodal Transformer and the classification head are updated, while the encoders remain frozen. This freezing strategy is structurally essential to prevent feature collapse. Since the Multimodal Transformer is initialized with random weights, joint fine-tuning would generate large, noisy gradients during early backpropagation. These unstable gradients risk distorting the delicate fine-grained alignment established in Stage 1 before the fusion module converges. By freezing the encoders, we force the Transformer to model complex dependencies based on a stable and highly discriminative embedding geometry.

### 3.4. Datasets

**EEG-ImageNet.** The EEG-ImageNet dataset [11] collects EEG data from 16 participants who viewed 4000 images selected from the ImageNet dataset [32]. It is a recently released EEG-image dataset designed for mixed-granularity visual recognition. The stimulus set contains 80 categories, including 40 coarse-grained and 40 fine-grained categories, with 50 images per category. 50 images of each category are presented sequentially. Each image is displayed for 500 ms, followed by a short fixation interval. The corresponding neural responses are recorded by a 62-channel EEG system with a sampling rate of 1000 Hz. This diverse labelling scheme facilitates the study of broad and specific neural representations associated with visual stimuli. This category-wise presentation order should be considered when interpreting EEG decoding results, as temporal continuity or block-related structure may affect EEG-based classification if not explicitly controlled.

**EEGCVPR.** The EEGCVPR Dataset [5] consists of electroencephalogram (EEG) recordings collected from six healthy participants who were instructed to view a series of visual stimuli selected from a subset of the ImageNet dataset, comprising 2000 images across 40 object categories, with 50 images per category. During data acquisition, each image was presented for 0.5 s, and participants were allowed a 10-s rest period after viewing all 50 images within the same category. The corresponding neural responses were recorded using a 128-channel EEG system with a sampling rate of 1000 Hz. For preprocessing, a temporal window between 40 ms and 480 ms after stimulus onset was selected to ensure that the extracted EEG segment primarily captured cortical responses elicited by the current visual stimulus while minimising residual effects from the preceding image.

### 3.5. EEG Preprocessing and Evaluation Protocol

For EEG-ImageNet, we used the officially released EEG-image pairs. According to the dataset protocol [11], the raw EEG recordings were preprocessed by the dataset providers using offline linked-mastoids re-referencing, 0.5–80 Hz band-pass filtering, 50 Hz environmental noise removal, and artifact removal for abnormal amplitudes, blinks, and head movements. Each released EEG sample corresponds to the 500 ms image-presentation window. Following the benchmark feature-extraction protocol, we used the 40–440 ms segment of each EEG trial as the model input to reduce the influence of adjacent visual stimuli.

For EEGCVPR, we followed the preprocessing protocol reported in the original dataset publication [5]. The EEG recordings were processed using a 49–51 Hz notch filter and a 14–71 Hz second-order Butterworth band-pass filter. The first 40 ms after stimulus onset were discarded, and the subsequent EEG segment was retained to focus on cortical responses elicited by the current visual stimulus. The released EEGCVPR data used in prior work contain valid EEG-image pairs after the removal of bad samples.

No additional trial rejection was applied beyond the preprocessing and bad-sample removal already performed in the released datasets. To make the effective sample size explicit, we define one EEG-image pair as one visual stimulus presentation paired with the corresponding EEG segment from one participant. According to the EEG-ImageNet dataset paper and the released metadata used in this study, EEG-ImageNet contains 63,850 EEG-image pairs from 16 participants. For EEGCVPR, the recording protocol included 12,000 expected image-response segments (6 subjects × 2000 images), of which 36 were excluded by the dataset providers because of low recording quality or lack of visual fixation, leaving 11,964 retained EEG-image pairs. For the main within-dataset experiments, EEG-ImageNet and EEGCVPR were divided into training, validation, and test sets using a fixed stratified 8:1:1 split. For the main random within-dataset EEG-ImageNet protocol, where each category contains 50 stimulus images, this corresponds to 40 stimulus images for training, five stimulus images for validation, and five stimulus images for testing within each category; the released EEG samples associated with these stimuli were assigned to the corresponding subset. The same preprocessing, split protocol, and evaluation settings were used for all compared baselines and ablation variants. Reported means and standard deviations were computed over five runs using pre-specified random seeds under the same fixed split protocol. For the cross-subject experiment on EEG-ImageNet, Subjects 1–12 were used as the source training group and Subjects 13–16 were held out as unseen target subjects, without subject-specific calibration.

To further assess the possible influence of category-wise stimulus ordering and the associated temporal-continuity confound, we additionally constructed a temporally separated split on EEG-ImageNet according to the within-category presentation order. In this control protocol, images and their corresponding EEG trials were partitioned by their order within each category block: the first 40 stimuli were used for training, the next five for validation, and the final five for testing. Thus, no EEG-image pair from the held-out later temporal segment was used for model fitting. This protocol was used only as an additional leakage-control analysis and was not used for hyperparameter selection. We also performed a class-preserving mismatched-EEG control, in which the image and class label were kept unchanged but the EEG input was replaced by another EEG trial from the same class (j≠i). This control tests whether the final gain depends on the correctly paired EEG-image trial rather than only on class-level or block-level EEG nuisance information.

### 3.6. Implementation Details

Our framework was implemented using PyTorch 2.1.0 on NVIDIA RTX 3090 GPUs. For each evaluation protocol, all models were trained over five matched runs using five pre-specified random seeds. The data split was kept fixed within each protocol, while model initialization, mini-batch ordering, and stochastic data augmentation varied with the seed.

**Stage 1: Representation Alignment.** We utilised a dual-encoder architecture. The EEGConformer, processing 62 channels and 400 time-points with a patch size of 10, comprises a 6-layer Transformer encoder (embedding dim = 256, 8 heads, feed-forward dim = 1024, dropout = 0.3). The visual encoder employs a pre-trained ResNet-101 with a frozen backbone, followed by a learnable linear layer projecting the 2048-dimensional features to 256 dimensions. Both modalities are mapped to a 128-dimensional shared space via a projection MLP and subsequently $L_{2}$-normalised. We trained this stage for 200 epochs using the Adam optimiser (learning rate $3\times 10^{-4}$, weight decay $1\times 10^{-5}$) with a batch size of 64. Data augmentation strategies included standard random resized cropping (224×224) and horizontal flipping for images, while random Gaussian noise (σ=0.05) was injected into the EEG signals to prevent overfitting to signal artefacts. For the Stage 1 contrastive objective, the temperature was set to τ=0.1, and the intra-modality loss weights were set to $\beta_{eeg}=1.0$ and $\beta_{img}=1.0$. For Hard Negative Weighting, we used $\lambda_{h}=1.0$ and k=10 as the main setting. These values were kept fixed across the main experiments unless otherwise specified in the sensitivity analysis.
**Stage 2: Fusion and Classification.** The pre-trained and aligned encoders were frozen to preserve their learned high-quality representations. The aligned feature vectors were fed into our multimodal Transformer (L=2 blocks, 8 attention heads), and the resulting fused representation was passed to a two-layer MLP classification head (128 hidden units, dropout = 0.3). This stage was trained for 100 epochs with a batch size of 16. We employed the Adam optimiser with a higher learning rate of $1\times 10^{-3}$ and a weight decay of $1\times 10^{-4}$, optimising the standard cross-entropy loss with label smoothing (ε=0.1). To enhance robustness, we applied a more aggressive set of data augmentations specifically to the visual modality, including random resized cropping, rotation ($\pm 15^{\circ }$), colour jittering, and random erasing.

To evaluate the overall effectiveness of our proposed “Align and Fuse” framework, we employed multiple quantitative metrics, including Top-1 Accuracy, Recall, Precision, and F1-Score, to comprehensively assess both the overall classification capability and class-level balance. To improve the stability and reliability of our results, all experiments were conducted over the same five matched random seeds, and we report the mean and standard deviation across these runs.

## 4. Results

### 4.1. Main Results and Analysis

Before presenting the same-protocol results, we first summarize representative EEG-image visual recognition and CLIP-style neuro-visual methods in Table 1. These methods contextualize representative task settings in the EEG-image literature, including standard classification, zero-shot recognition, and contrastive EEG-image representation learning. To avoid presenting heterogeneous results as a single leaderboard, Table 1 groups entries by task type and dataset protocol. Because these methods differ in datasets and evaluation protocols, the reported values are best read as task-specific contextual references rather than strictly identical-protocol comparisons. For example, the 96.17% result reported by Cudlenco et al. was obtained on an author-collected dataset, whereas our 95.82% result is reported on the public EEGCVPR benchmark. Same-protocol comparisons with the Cudlenco-style fusion baseline are provided in the main EEG-ImageNet and EEGCVPR result tables below.

We conducted comprehensive evaluations on the EEG-ImageNet and EEGCVPR benchmarks by comparing our framework against three categories of baselines: Vision-Only, EEG-Only, and Multimodal Fusion. For the Vision-Only setting, we employed widely used deep CNNs pre-trained on ImageNet, including ResNet [2], GoogLeNet [34], DenseNet [35], and Inception-v3 [36]. The EEG-Only baseline utilises the EEGConformer encoder to capture temporal neural dynamics. To assess the contribution of our alignment module, we implemented two multimodal baselines: the fusion method by Cudlenco et al. [6], which directly concatenates independent features, and a rigorous ‘Standard Transformer’ baseline. The latter utilises the exact same dual-stream architecture as our framework but is trained end-to-end without the proposed Stage 1 alignment. This isolation allows us to quantify the specific gain derived from our representation alignment strategy. The quantitative results are summarised in Table 2.

Table 2 reports the original stratified random split on EEG-ImageNet. Under this protocol, our full “Align and Fuse” framework achieves a Top-1 accuracy of 92.56 ± 0.22%, outperforming the Vision-Only baseline (89.17 ± 0.21%) by 3.39 percentage points. This substantial margin may indicate that visual features alone face limitations when dealing with texture-biased or visually ambiguous categories. The inclusion of EEG signals helps alleviate this limitation by acting as a complementary cue that provides stimulus-related neural information not fully captured by the visual backbone. Furthermore, our framework outperforms the naive concatenation baseline [6] (90.15 ± 0.34%) by 2.41 percentage points, suggesting that the “semantic gap” between unaligned heterogeneous spaces can impede effective fusion. Crucially, while the ‘Standard Transformer’ serves as a strong baseline (91.24 ± 0.29%) due to its attention mechanism, it still lags behind our framework by 1.32 percentage points. This performance gap supports the interpretation that jointly learning alignment and fusion can be a difficult optimisation problem. Without the explicit geometric constraints imposed by our Stage 1 contrastive learning, the Transformer may struggle to simultaneously align distributions and classify features. By decoupling these tasks—learning an aligned geometry first, then reasoning upon it—our framework allows the fusion module to focus purely on modelling complex inter-modal dependencies. To further assess robustness, we extended the evaluation to the EEGCVPR dataset (Table 3), observing a consistent pattern where our framework (95.82 ± 0.19%) surpasses both the best Vision-Only model (Inception-v3, 92.95 ± 0.20%) and the ‘Standard Transformer’ (95.10 ± 0.24%). These consistent gains across datasets with differing characteristics suggest that our proposed alignment strategy remains effective under different dataset conditions.

To evaluate real-world applicability, we employed a challenging cross-subject held-out protocol, partitioning the 16 EEG-ImageNet participants into a training source group (Subj. 1–12) and a testing target group (Subj. 13–16). The evaluation was conducted in a strict calibration-free manner, and all cross-subject baselines were evaluated under the same protocol. As summarised in Table 4, the EEG-only EEGConformer baseline obtains an average Top-1 accuracy of 35.11 ± 1.52%, indicating that EEG-only decoding is strongly affected by inter-subject variability. The Standard Transformer baseline improves over the Vision-Only baseline (90.02 ± 0.23% vs. 89.17 ± 0.21%), suggesting that EEG information can still provide useful complementary cues under subject shift. Our full framework further improves the average accuracy to 90.92 ± 0.31%, suggesting that the proposed alignment stage provides additional benefit over direct multimodal fusion without subject-specific calibration.

### 4.2. Leakage-Control and EEG-Pairing Control Analysis

Because both EEG-ImageNet and EEGCVPR contain category-wise stimulus blocks, we conducted additional control experiments on EEG-ImageNet to examine whether the observed EEG-augmented gains could be attributed mainly to temporal continuity, block identity, or non-specific EEG nuisance information. Table 5 summarizes two complementary analyses. First, we evaluated the models under a temporally separated split constructed according to the within-category presentation order. Second, we replaced the correctly paired EEG trial with another trial from the same class to form a class-preserving mismatched-EEG control. We further added a fully label-shuffled control in which the EEG input was drawn from a different class under the same temporal-split evaluation protocol.

As an additional robustness analysis, we also repeated the temporal-split control on EEGCVPR, and the results are summarized in Table 6. Because EEGCVPR presents the 50 images of each class consecutively, we constructed the split at the image level within each class block: the first 40 images were assigned to training, the next five to validation, and the final five to testing. All retained EEG segments associated with the same image followed the same split assignment.

Under this EEGCVPR temporal split, the EEG-only model decreased from 63.46% under the random split to 42.15%, indicating that EEG-only decoding on EEGCVPR is also sensitive to block/order structure. The Vision-only baseline remained strong at 92.86%, while the Standard Transformer and our fusion model reached 93.18% and 93.50%, respectively. Thus, the temporal-split gain on EEGCVPR was modest (+0.64 percentage points over Vision-only and +0.32 points over the Standard Transformer), but remained positive under a temporally separated protocol.

Under the temporally separated split, which we treat as the primary controlled EEG-ImageNet result in this revision, the EEG-only model decreased from 45.50% to 27.85%, indicating that EEG-only decoding can be affected by temporal or block-related structure in the original sample-level protocol. However, the EEG-only temporal-split accuracy remained well above the 80-class chance level of 1.25%, suggesting that the EEG signal still contains decodable stimulus-related information. This pattern indicates that EEG features are not sufficiently reliable as a standalone 80-class classifier under the stricter split, but they can still act as weak complementary cues when fused with strong visual representations. More importantly, our full framework maintained a positive gain under the temporal split, improving over both the Vision-only baseline (91.12% vs. 89.08%) and the Standard Transformer baseline (91.12% vs. 89.95%).

The same-class mismatched-EEG control further clarifies the role of correct EEG-image pairing. Replacing the matched EEG trial with another EEG trial from the same class reduced Top-1 accuracy from 91.12% to 90.25%. Thus, of the 2.04 percentage-point gain of matched fusion over the Vision-only baseline, 1.17 percentage points, or approximately 57%, remain when the EEG is replaced by another trial from the same class. This indicates that a substantial portion of the gain is not strictly trial-specific and may reflect class-level, block-level, or other non-trial-specific EEG information. The matched condition adds 0.87 percentage points beyond the same-class mismatched condition, which we interpret as a more limited trial-pairing-specific contribution under this control. In the fully different-class shuffled control, performance decreased to 87.65% Top-1 accuracy and 87.82% macro-F1, falling below the Vision-only baseline. This indicates that class-inconsistent EEG does not explain the fusion gain and may instead introduce conflicting information. Together, these controls provide a more conservative assessment of the EEG contribution under stricter evaluation conditions and suggest that the observed gain should not be interpreted as purely trial-specific EEG benefit.

### 4.3. Statistical Significance Analysis

To assess whether the main improvements were stable across matched runs, we performed paired *t*-tests over the five random seeds used for each corresponding comparison. Holm–Bonferroni correction was applied across the five planned comparisons in Table 7. We report the run-level mean difference in percentage points of Top-1 accuracy to summarize the practical magnitude of each comparison, together with paired-sample Cohen’s $d_{z}$ as the effect-size measure.

All five planned comparisons remained significant after Holm–Bonferroni correction. These tests provide run-level evidence that the main improvements are stable across the matched training seeds, with mean differences ranging from 0.71 to 2.04 percentage points and paired-sample effect sizes ranging from $d_{z}=2.89$ to $d_{z}=4.77$. These statistics are computed from the same five fixed-seed runs used to report the corresponding mean and standard deviation. Because they are based on five matched seeds, we interpret them as supportive run-level evidence rather than population-level effect-size estimates.

### 4.4. Performance Analysis on Fine-Grained Categories

While the overall metrics in Table 2 establish the framework’s superiority, the true test of neuro-visual decoding lies in its ability to resolve ambiguities. A fundamental bottleneck in mixed-granularity recognition lies in distinguishing highly similar categories where visual features alone are often insufficient due to subtle inter-class variance. To rigorously evaluate our framework’s capability in this regard, we conducted a comprehensive analysis combining quantitative metrics and qualitative visualisations.

**Figure 3:**
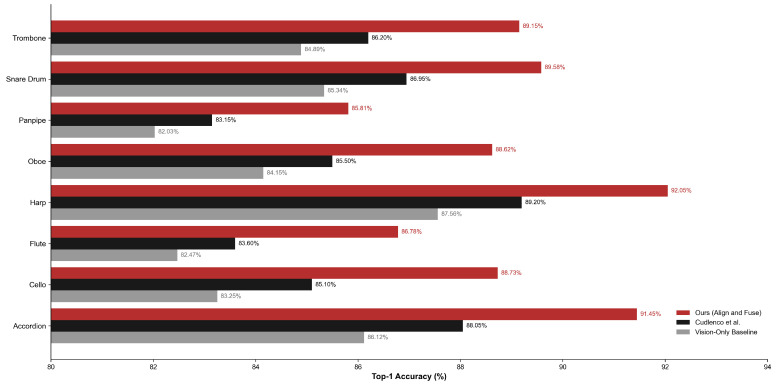
Performance comparison on a challenging subset of fine-grained musical instruments. The bar chart contrasts the Top-1 Accuracy of the Vision-Only baseline (grey), the multimodal fusion method by Cudlenco et al. [6] (black), and our “Align and Fuse” framework (red), highlighting consistent performance gains across these visually similar categories.

First, we examined the impact on per-class accuracy using a semantically challenging subset of the EEG-ImageNet dataset: Musical Instruments. As illustrated in Figure 3, categories such as the Panpipe, Flute, and Cello exhibit dense visual similarities—characterised by isomorphic tubular structures and metallic textures—causing the Vision-Only baseline to struggle, yielding an average accuracy of approximately 84%. In contrast, our “Align and Fuse” framework achieves consistent improvements across this entire group. Figure 3 further highlights that while the multimodal fusion method proposed by Cudlenco et al. [6] improves upon the Vision-Only baseline, its gains are often limited on highly ambiguous categories. For instance, on the Panpipe, Cudlenco et al. yields a marginal improvement of 1.12% (from 82.03% to 83.15%). In sharp contrast, our framework achieves a substantial boost to 85.81%. Similarly, for the Flute, our method outperforms Cudlenco et al. by 3.18% (86.78% vs. 83.60%). Even on structurally complex objects such as the Accordion, we observe a substantial gain of over 5% compared to the visual baseline.

**Figure 4:**
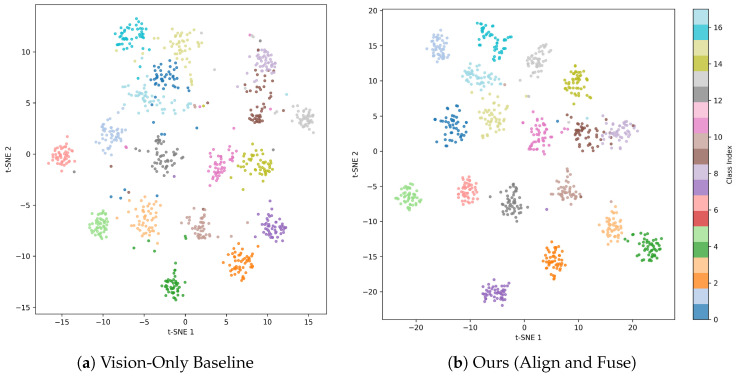
Qualitative comparison of feature embedding spaces using t-SNE on a representative mixed-granularity subset of 18 categories. (**a**) Vision-Only baseline. (**b**) Our “Align and Fuse” framework. **Indices 0–7** correspond to *Musical Instruments* (e.g., Flute, Cello), **Indices 11–13** to *Dog Breeds*, and the remaining indices to coarse-grained anchors.

To understand the geometric mechanism behind these improvements, we visualised the feature embedding spaces using t-SNE on a representative mixed-granularity subset (including both instruments and dog breeds), as shown in Figure 4. Comparison between Figure 4a,b reveals that the Vision-Only baseline suffers from severe entanglement in fine-grained clusters, particularly in the blue and cyan regions corresponding to Musical Instruments (Indices 0–7). However, our framework effectively disentangles these hard negatives, forming compact and well-separated clusters. This visualization suggests that the accuracy gains are associated with a more discriminative feature space learned via our Hard Negative Weighting strategy.

**Figure 5:**
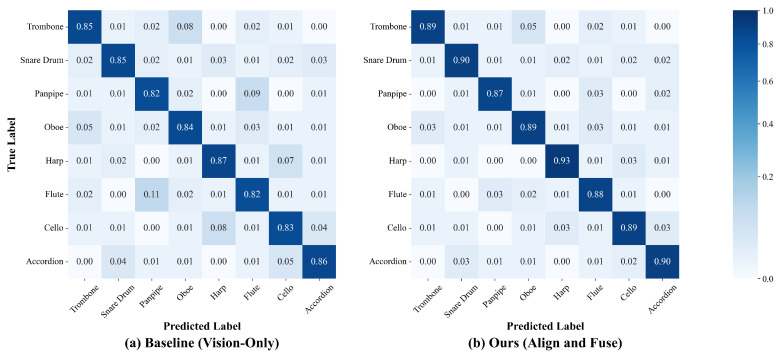
Visualisation of normalised confusion matrices on a challenging fine-grained subset. The heatmaps contrast the classification performance of (**a**) the Vision-Only baseline and (**b**) our “Align and Fuse” framework, highlighting the reduction of misclassifications between visually similar categories.

Finally, to investigate the error distribution at a granular level, we visualised the normalised confusion matrices for this subset (Figure 5). The Vision-Only baseline (Figure 5a) exhibits significant confusion between structurally similar objects due to the lack of semantic guidance. Notably, 9% of Panpipe samples are misclassified as Flute, and 8% of Cello samples are confused with Harp, suggesting that visual features alone are insufficient to resolve such fine-grained ambiguities. However, our framework (Figure 5b) effectively suppresses these specific off-diagonal misclassifications. The confusion between Panpipe and Flute is drastically reduced to 3%, and the misclassification of Cello as Harp drops to 3%.

These results provide empirical support that our Hard Negative Weighting mechanism encourages the model to learn subtle, category-defining features. By leveraging aligned EEG signals as complementary cues, our approach helps disentangle visually ambiguous patterns, thereby surpassing conventional direct fusion paradigms.

### 4.5. Ablation Study

To systematically evaluate the contribution of each core component, we performed a comprehensive ablation study on the EEG-ImageNet dataset (Table 8). We examined variants by progressively adding multimodal fusion and then changing only the Stage 1 alignment objective, while keeping the encoders, downstream Transformer fusion stage, and evaluation protocol unchanged.

**Effect of Multimodal Transformer Fusion.** We first assessed the fusion mechanism without explicit alignment. Introducing the Multimodal Transformer (Model 2, corresponding to the ‘Standard Transformer’ baseline) boosts Top-1 accuracy from 89.17 ± 0.21% to 91.24 ± 0.29%. This indicates that the co-attention mechanism possesses an implicit capability to model cross-modal dependencies. However, the performance is still capped by the semantic gap between modalities. Without prior alignment, the attention mechanism is forced to learn correlations from scratch on unaligned distributions, limiting its ability to resolve fine-grained distinctions effectively.

**Impact of Contrastive Alignment Objectives.** The comparison among Models 2–5 further clarifies how the alignment objective affects recognition performance. Adding CLIP-style paired EEG-image contrastive alignment improves Top-1 accuracy from 91.24 ± 0.29 to 91.52% ± 0.27%, suggesting that paired cross-modal alignment provides a useful shared representation. Replacing paired instance-level alignment with standard supervised contrastive learning further improves performance to 91.85 ± 0.26%, indicating that class-level positive sets are beneficial in this supervised recognition setting. Finally, adding Hard Negative Weighting increases the accuracy to 92.56 ± 0.22% (+0.71% over standard SCL). Because Models 4 and 5 share the same supervised contrastive formulation except for the hard-negative weighting term, this comparison isolates the contribution of explicitly emphasizing confusable negative samples. These results support the interpretation that hardness-aware alignment is beneficial for constructing a more discriminative geometry in mixed-granularity neuro-visual recognition.

To further examine whether the benefit of HNW depends on a narrow hyperparameter choice, we conducted a one-parameter-at-a-time sensitivity analysis for the weighting strength $\lambda_{h}$ and the sigmoid slope *k*. For the $\lambda_{h}$ sweep, *k* was fixed to 10; for the *k* sweep, $\lambda_{h}$ was fixed to 1.0. Other loss hyperparameters were kept unchanged.

As shown in Table 9, setting $\lambda_{h}=0$ reduces the objective to standard SCL and yields lower performance than all tested HNW variants. Moderate weighting ($\lambda_{h}=1.0$) achieved the best result, whereas a stronger setting ($\lambda_{h}=1.5$) produced a slight decrease, suggesting that overly strong hard-negative emphasis may introduce unnecessary repulsion or optimization noise. A similar trend was observed for *k*: a smoother gating function (k=5) was less effective, while a steeper setting (k=15) slightly reduced performance relative to the main setting. Overall, the results indicate that HNW is beneficial within the evaluated range and is not dependent on a single fragile hyperparameter choice. The values in this sensitivity table are computed from the same five fixed-seed runs used to report the mean and standard deviation. At the same time, stronger weighting is not necessarily better.

### 4.6. Generality and Robustness Analysis

To demonstrate that the effectiveness of our “Align and Fuse” framework is not confined to specific network architectures, we conducted extensive generalisation experiments. We systematically evaluated the framework’s robustness by varying both the visual backbones and the EEG encoders, ensuring that our reported gains stem from the proposed alignment strategy rather than a specific combination of modules.

**Generality Across Visual Backbones.** We applied our framework to three additional widely-used CNN architectures: GoogLeNet, Inception-v3, and DenseNet-121, replacing the ResNet-101 backbone while keeping the EEGConformer fixed. The results in Table 10 reveal a consistent performance boost across all architectures. Notably, improvements are observed in both lighter networks (GoogLeNet, +2.85%) and highly sophisticated ones (DenseNet, +3.79%). This universality suggests that EEG-derived stimulus-related cues may provide complementary information beyond pixel-level visual features, even when stronger visual extractors are used. Our framework effectively bridges this gap, suggesting that the alignment strategy is a generalizable enhancer compatible with various visual backbones.

**Impact of Different EEG Encoders.** To assess whether our framework relies solely on the Transformer-based EEG encoder, we replaced the EEGConformer with two classic architectures: a bi-directional LSTM and EEGNet. As shown in Table 11, while the EEGConformer yields the highest performance due to its global attention mechanism, other encoders also bring consistent improvements (e.g., +2.15% with EEGNet). These results demonstrate that our “Align and Fuse” strategy is encoder-agnostic. The performance gains are driven by the effective alignment of feature spaces rather than the specific architecture used to extract them, supporting the broad applicability of our method across different neural encoders.

### 4.7. Visual Analysis

**Figure 6:**
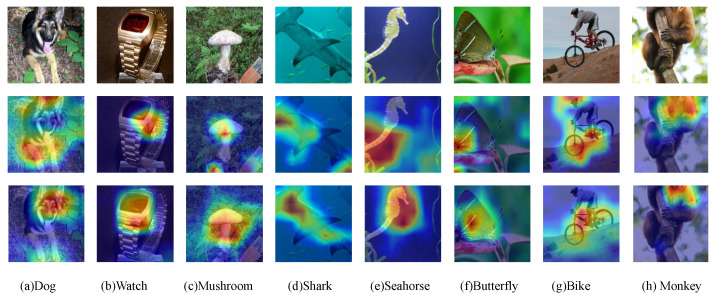
CAM visualisation for examples in the validation set. The first row shows the original images, and the second and third rows show the CAMs of the Vision-Only baseline and our “Align and Fuse” framework, respectively.

To provide qualitative insights into the mechanisms underlying our framework’s performance, we conducted a cross-modal saliency analysis using Grad-CAM [37]. As illustrated in Figure 6, the Vision-Only baseline (middle row) frequently exhibits a lack of semantic focus, indiscriminately activating on environmental contexts—such as grass or water—rather than the object’s core structure. This reflects the typical data-driven bias of CNNs, which correlate background statistics with class labels.

In sharp contrast, our “Align and Fuse” framework (bottom row) demonstrates a shift toward semantically relevant image regions. By incorporating aligned EEG signals as complementary cues, the model modulates its attention distribution. For example, in the case of the Shark (Figure 6d) and Butterfly (Figure 6f), our model tightly contours the subject while suppressing the surrounding water or foliage that confused the baseline. Furthermore, for objects with complex geometries like the Bike (Figure 6g), the aligned representations enable the model to disentangle the object from its support (e.g., tree branches), placing greater emphasis on discriminative parts like the mechanical frame. These saliency maps suggest that our strategy may guide the model toward attention patterns that are more consistent with semantically relevant visual cues. Since the ResNet-101 visual backbone remains frozen in our framework, these Grad-CAM differences reflect the downstream fusion Transformer and classification head reweighting frozen visual feature maps under the influence of aligned EEG features.

### 4.8. Temporal Analysis of EEG Contributions

To characterize how EEG information contributes across time, we conducted an occlusion-based temporal analysis of the EEG input. During inference, different temporal segments of the EEG input were masked while keeping the image input, model parameters, and evaluation protocol unchanged. The full EEG input covered the 40–440 ms post-stimulus interval, and four temporal windows were evaluated: 40–120 ms, 120–200 ms, 200–300 ms, and 300–440 ms.

As shown in Table 12, masking any temporal segment led to a performance decrease, indicating that the framework uses EEG information distributed across the 40–440 ms interval. The largest drop was observed when masking the 200–300 ms window (1.30%), followed by the 120–200 ms window (0.98%). These results suggest that mid-latency EEG responses within 120–300 ms contribute more strongly to the EEG-augmented recognition process than the earliest or latest segments.

## 5. Discussion

In this paper, we introduced “Align and Fuse,” a novel two-stage framework designed to effectively integrate human neural signals with deep learning models for enhanced visual recognition, particularly for complex, mixed-granularity tasks. Our work systematically addresses two key bottlenecks in the field: learning fine-grained, class-discriminative representations and achieving a deep, synergistic fusion of multimodal features. The current framework requires an EEG recording corresponding to the viewed stimulus at inference time; therefore, it should be interpreted as an EEG-augmented human-in-the-loop recognition framework rather than a standalone image-only recognition system.

Our primary contribution lies in a principled two-stage paradigm. First, we proposed a Multimodal Supervised Contrastive Learning objective enhanced by a Hard Negative Weighting mechanism. This design refines decision boundaries for confusable categories, ensuring that the learned representations are not only modality-aligned but also semantically distinct. Second, we introduced a decoupled fusion strategy with a Multimodal Transformer. By freezing the aligned encoders, we enable the fusion module to focus on modelling complex neuro-visual interactions while preserving the representation geometry learned during the alignment stage.

**Scalability and training stability.** The proposed Hard Negative Weighting is computed from pairwise similarities within each mini-batch rather than from exhaustive pairwise comparisons over the entire image collection. Therefore, its direct computational cost is mainly controlled by the batch size used for contrastive alignment. In our experiments, the sigmoid gating parameters were kept fixed across datasets, which avoids dataset-specific hyperparameter search and supports stable training under the evaluated settings. Nevertheless, when the method is scaled to datasets with substantially larger semantic spaces or thousands of fine-grained categories, the density of hard negatives may increase. In such cases, memory-bank strategies, approximate hard-negative mining, or curriculum-based sampling could be incorporated to maintain efficient and stable optimization.

**Negative transfer and alignment geometry.** Direct EEG-image vector alignment may introduce a risk of transferring modality-specific neural noise into the shared representation space. Our two-stage design partly mitigates this issue by separating representation alignment from multimodal decision fusion. Stage 1 constructs a shared geometry between EEG and image embeddings, whereas Stage 2 freezes the aligned encoders and trains only the fusion Transformer and classifier. This design reduces the chance that noisy gradients from the fusion stage distort the aligned representation space. Even so, point-to-point alignment should not be viewed as the only possible solution. Future work could incorporate topology-preserving or distribution-level constraints to better maintain the relational structure among samples and further reduce negative transfer across modalities.

**Complementarity and class-level EEG cues.** The visual-backbone experiments indicate that stronger visual extractors improve the baseline recognition performance, but EEG-augmented fusion still provides consistent gains across different CNN backbones. This pattern is consistent with the view that EEG-derived features may provide complementary stimulus-related information rather than simply duplicating low-level visual features. However, the backbone results also admit a more conservative interpretation: because weaker visual backbones receive larger relative gains and the EEG-augmented variants converge into a narrower accuracy band, the EEG branch may also supply a relatively stable class-level or block-level cue that benefits weaker image models more strongly. Thus, complementarity is a plausible but not exclusive interpretation. Similarly, the EEG-encoder experiments show that the framework does not rely exclusively on EEGConformer, although the Transformer-based EEG encoder offers the strongest performance. Overall, these one-factor-at-a-time analyses support combined and cumulative contributions from EEG feature extraction, discriminative visual encoding, and hardness-aware alignment, but they do not by themselves establish a statistical interaction effect among components.

**Limitations, applications, and future work.** Several limitations should be acknowledged. First, although EEG-ImageNet is larger than earlier EEG-image benchmarks, the number of EEG participants is still limited, and stronger evidence is needed to assess subject variability in broader populations. Second, both EEG-ImageNet and EEGCVPR are collected under controlled visual-stimulus settings, so dataset bias and the gap to naturalistic visual environments remain important concerns. In particular, EEG-ImageNet uses a category-wise stimulus presentation design, and EEGCVPR also presents the 50 images within each category as a block. Such designs are useful for controlled EEG acquisition but may introduce temporal-continuity, slow-drift, or block-related effects in EEG decoding. This concern has been explicitly raised in prior analyses of block-design EEG classification experiments [38], although subsequent work argued that appropriate filtering, control conditions, and experimental design choices can mitigate temporal-correlation bias [39]. The additional temporal-split and mismatched-EEG controls reduce the possibility that our gains are solely driven by these effects, but they should be viewed as control analyses rather than a complete removal of all dataset-level confounds. Future EEG-image benchmarks would benefit from more randomized or counterbalanced stimulus presentation protocols to further separate stimulus-related EEG responses from temporal-order effects. Third, the temporal-window occlusion analysis provides supportive but limited evidence that mid-latency EEG responses within 120–300 ms are more influential under the original split. Because the largest observed drop is modest and the analysis was not repeated under the temporal split, this result should be interpreted as exploratory rather than as definitive neural-mechanistic evidence. Future work should further examine temporal-split occlusion, EEG-side channel-level importance, and frequency-band relevance to clarify which neural dynamics drive cross-modal alignment. Finally, because the current model consumes EEG features during inference, real-time deployment would require additional advances in synchronized EEG acquisition, low-latency inference, online noise handling, and calibration-free cross-subject adaptation. Despite these limitations, the framework may support future human-in-the-loop settings in which EEG can be measured during inference, such as BCI-assisted visual inspection, neuroergonomic decision support, assistive communication, and clinical cognitive assessment. These applications should be interpreted as future directions under EEG-available inference conditions rather than immediate standalone vision deployment claims.

## 6. Conclusions

This study presented “Align and Fuse,” a two-stage Transformer-based framework that effectively integrates EEG signals with visual representations for mixed-granularity recognition. By combining hardness-aware multimodal contrastive learning with a decoupled multimodal Transformer fusion stage, the framework achieves 91.12% Top-1 accuracy under the temporally separated EEG-ImageNet control protocol and 92.56% under the original stratified random split; it also achieves 95.82% on the 40-class EEGCVPR dataset. Cross-subject experiments and Grad-CAM analyses provide additional evidence for the framework’s robustness and its capacity to shift visual attention toward semantically relevant regions. Together with the additional temporal-split and mismatched-EEG controls, these results highlight the potential of human-in-the-loop EEG-augmented recognition, while future work should further validate the framework under more randomized and naturalistic EEG-image protocols.

## Figures and Tables

**Table 1:** Performance comparison with representative EEG-image and CLIP-style neuro-visual methods. Entries are grouped by task type and dataset protocol; therefore, reported values should be read within each block and not as a same-protocol leaderboard across blocks.

Method	Dataset	Task/Setting	Reported Result
*(A) Public-Dataset Closed-set EEG-Visual Reports*			
Palazzo et al. [20]	EEGCVPR	Joint EEG-visual learning	94.40%
**Ours (Align and Fuse)**	**EEGCVPR**	**E + V closed-set classification**	**95.82%**
*(B) Author-Collected Closed-set EEG-Visual Reports*			
Cudlenco et al. [6]	Author-collected	E + V classification	96.17%
*(C) Zero-shot EEG-Based Recognition/Retrieval*			
Singh et al. (EEGClip) [22]	EEGCVPR	Image zero-shot setting	95.00%
NICE-EEG (NICE-GA) [23]	THINGS-EEG2 [33]	200-way zero-shot recognition	15.60%

**Table 2:** Performance comparison on the 80-class EEG-ImageNet dataset. Results are reported as mean ± standard deviation over five fixed-seed runs.

Type	Method	Top-1 Acc	Precision	Recall	F1
I (Image-only)	ResNet-101	89.17 ± 0.21	90.13 ± 0.24	88.87 ± 0.26	89.48 ± 0.22
	GoogLeNet	88.30 ± 0.28	87.69 ± 0.31	89.03 ± 0.27	88.35 ± 0.25
	Inception-v3	89.14 ± 0.25	88.95 ± 0.27	88.07 ± 0.29	88.51 ± 0.24
	DenseNet	88.01 ± 0.30	86.92 ± 0.33	86.93 ± 0.31	86.92 ± 0.29
E (EEG-only)	EEGConformer	45.50 ± 0.83	46.82 ± 0.88	45.35 ± 0.91	46.07 ± 0.85
I + E (Multimodal)	Cudlenco et al. [6]	90.15 ± 0.34	90.05 ± 0.36	90.35 ± 0.33	90.18 ± 0.32
	Standard Transformer	91.24 ± 0.29	91.35 ± 0.30	91.15 ± 0.32	91.23 ± 0.28
	**Ours (Align and Fuse)**	**92.56 ± 0.22**	**92.75 ± 0.24**	**92.51 ± 0.25**	**92.60 ± 0.23**

**Table 3:** Performance comparison on the 40-class EEGCVPR dataset. Results are reported as mean ± standard deviation over five fixed-seed runs.

Type	Method	Top-1 Acc	Precision	Recall	F1
I (Image-only)	ResNet-101	92.75 ± 0.18	93.35 ± 0.21	93.02 ± 0.20	93.18 ± 0.19
	GoogLeNet	90.64 ± 0.26	91.10 ± 0.28	90.68 ± 0.27	90.89 ± 0.25
	Inception-v3	92.95 ± 0.20	93.25 ± 0.22	92.70 ± 0.24	92.97 ± 0.21
	DenseNet	91.55 ± 0.24	91.85 ± 0.26	92.03 ± 0.25	91.94 ± 0.23
E (EEG-only)	EEGConformer	63.46 ± 0.71	64.25 ± 0.75	63.10 ± 0.78	63.67 ± 0.72
I + E (Multimodal)	Cudlenco et al. [6]	94.51 ± 0.28	94.60 ± 0.30	95.11 ± 0.27	94.88 ± 0.26
	Standard Transformer	95.10 ± 0.24	95.05 ± 0.25	95.32 ± 0.26	95.18 ± 0.23
	**Ours (Align and Fuse)**	**95.82 ± 0.19**	**95.95 ± 0.20**	**96.13 ± 0.21**	**96.04 ± 0.19**

**Table 4:** Cross-subject generalisation performance under the same held-out-subject protocol. Subjects 1–12 were used for training, and Subjects 13–16 were used as unseen target subjects. Results are reported as mean ± standard deviation over five fixed-seed runs.

Test Subject	EEG-Only (EEGConformer)	Vision-Only (ResNet-101)	Standard Transformer	Ours (Align and Fuse)
Subject 13 (Unseen)	33.52 ± 1.45	89.17 ± 0.21	89.92 ± 0.24	90.32 ± 0.38
Subject 14 (Unseen)	36.85 ± 1.68	89.17 ± 0.21	90.35 ± 0.28	91.52 ± 0.34
Subject 15 (Unseen)	34.12 ± 1.54	89.17 ± 0.21	89.88 ± 0.22	91.10 ± 0.36
Subject 16 (Unseen)	35.94 ± 1.72	89.17 ± 0.21	89.95 ± 0.25	90.75 ± 0.41
**Average (Unseen)**	**35.11 ± 1.52**	**89.17 ± 0.21**	**90.02 ± 0.23**	**90.92 ± 0.31**

**Table 5:** Leakage-control and EEG-pairing control experiments on EEG-ImageNet. Panel A evaluates temporal-split robustness. Panel B evaluates the difference between correctly paired EEG, same-class mismatched EEG, and different-class shuffled EEG. Values are reported as mean ± standard deviation over five fixed-seed runs.

Panel A. Temporal-Split Robustness				
**Model Variant**	**Evaluation Protocol**	**Top-1 Acc. (%)**	**F1-Score (%)**	
EEG-only (EEGConformer)	Random split	45.50 ± 0.83	46.07 ± 0.85	
EEG-only (EEGConformer)	Temporal split	27.85 ± 1.12	28.15 ± 1.24	
Vision-only (ResNet-101)	Temporal split	89.08 ± 0.23	89.41 ± 0.24	
Standard Transformer	Temporal split	89.95 ± 0.28	90.12 ± 0.26	
**Ours (Align and Fuse)**	**Temporal split**	**91.12 ± 0.32**	**91.25 ± 0.30**	
**Panel B. EEG-pairing controls**				
**Condition**	**EEG Input**	**Top-1 Acc. (%)**	**F1-Score (%)**	**Drop**
Matched fusion	correct pair	91.12 ± 0.32	91.25 ± 0.30	0.00
Mismatched control	same-class trial	90.25 ± 0.35	90.38 ± 0.33	0.87
Vision-only baseline	none	89.08 ± 0.23	89.41 ± 0.24	2.04
Shuffled control	different-class trial	87.65 ± 0.41	87.82 ± 0.44	3.47

**Table 6:** Additional temporal-split robustness control on EEGCVPR. The temporal split follows the within-class presentation order and assigns all EEG segments associated with the same image to the same subset. Results are reported as mean ± standard deviation over five matched seeds.

Model Variant	Evaluation Protocol	Top-1 Acc. (%)	F1-Score (%)
EEG-only (EEGConformer)	Random split	63.46 ± 0.72	63.67 ± 0.69
EEG-only (EEGConformer)	Temporal split	42.15 ± 1.15	42.48 ± 1.13
Vision-only (ResNet-101)	Temporal split	92.86 ± 0.26	93.26 ± 0.29
Standard Transformer	Temporal split	93.18 ± 0.56	93.25 ± 0.55
**Ours (Align and Fuse)**	**Temporal split**	**93.50 ± 0.63**	**93.61 ± 0.58**

**Table 7:** Paired significance tests for key comparisons. Mean difference is reported in percentage points of Top-1 accuracy to indicate the practical magnitude of each comparison. $p_{\mathrm{adj}}$ denotes Holm–Bonferroni corrected *p*-values across the five planned comparisons, and $d_{z}$ denotes the paired-sample Cohen’s effect size.

Comparison	Mean Diff.	*p*-Value	$p_{\mathrm{adj}}$	$d_{z}$
Ours vs. unaligned multimodal fusion	1.32	0.0017	0.0052	3.33
Ours vs. standard SCL	0.71	0.0030	0.0052	2.89
Ours temporal vs. Vision-only temporal	2.04	0.0004	0.0022	4.77
Ours temporal vs. Standard Transformer	1.17	0.0011	0.0044	3.76
Matched EEG vs. same-class mismatched EEG	0.87	0.0024	0.0052	3.05

**Table 8:** Ablation study on the EEG-ImageNet dataset, comparing the contributions of multimodal fusion and different contrastive alignment objectives. Results are reported as mean ± standard deviation over five fixed-seed runs. SCL denotes supervised contrastive learning, and HNW denotes Hard Negative Weighting.

Model Variant	Image	EEG	Alignment Objective	Top-1 Acc
(1) Vision-only	✓	—	—	89.17 ± 0.21
(2) Unaligned multimodal fusion	✓	✓	—	91.24 ± 0.29
(3) Paired contrastive alignment	✓	✓	CLIP-style InfoNCE	91.52 ± 0.27
(4) Supervised contrastive alignment	✓	✓	Standard SCL	91.85 ± 0.26
**(5) Hardness-aware alignment**	✓	✓	**SCL + HNW**	**92.56 ± 0.22**

**Table 9:** Sensitivity analysis of the Hard Negative Weighting hyperparameters on EEG-ImageNet. One parameter was varied at a time while the other HNW parameter was fixed at the main setting. Results are reported as mean ± standard deviation over five matched verification runs, whose per-seed values are provided in the Supplementary Materials.

Parameter Varied	Value	Top-1 Acc. (%)	F1-Score (%)
$\lambda_{h}$	0.0 (Standard SCL)	91.85 ± 0.28	91.91 ± 0.22
	0.5	92.21 ± 0.36	92.28 ± 0.37
	1.0 (Main)	**92.56 ± 0.24**	**92.60 ± 0.23**
	1.5	92.42 ± 0.34	92.48 ± 0.28
*k*	5	92.18 ± 0.37	92.24 ± 0.26
	10 (Main)	**92.56 ± 0.24**	**92.60 ± 0.23**
	15	92.36 ± 0.30	92.41 ± 0.26

**Table 10:** Performance improvement comparison of our method using different visual encoders (with EEGConformer as the fixed EEG backbone).

Visual Model	Baseline (%)	Ours (%)	Improvement
GoogLeNet	88.30	91.15	2.85
Inception-v3	89.14	92.48	3.34
DenseNet	88.01	91.80	3.79
**ResNet-101 (Ours)**	89.17	**92.56**	**3.39**

**Table 11:** Performance improvement comparison of our method using different EEG encoders (with ResNet-101 as the fixed visual backbone).

EEG Model	Baseline (%)	Ours (%)	Improvement
Bi-LSTM	89.17	90.85	1.68
EEGNet	89.17	91.32	2.15
**EEGConformer (Ours)**	89.17	**92.56**	**3.39**

**Table 12:** Performance comparison under different EEG temporal-window masking settings on the EEG-ImageNet dataset. Values are reported as mean ± standard deviation over five fixed-seed runs, and accuracy drop is computed relative to the unmasked baseline.

Masked EEG Window	Top-1 Acc. (%)	F1-Score (%)	Accuracy Drop (%)
Unmasked	92.56 ± 0.22	92.60 ± 0.23	0.00
40–120 ms	92.15 ± 0.24	92.18 ± 0.25	0.41
120–200 ms	91.58 ± 0.26	91.61 ± 0.25	0.98
200–300 ms	91.26 ± 0.29	91.28 ± 0.28	1.30
300–440 ms	91.92 ± 0.25	91.95 ± 0.26	0.64

## Data Availability

No new data were generated in this study. The data analyzed in this work are publicly available from the EEGCVPR and EEG-ImageNet datasets, as described in the cited original publications. The implementation code, preprocessing scripts, training configurations, evaluation protocols, and trained checkpoints for the main reported models will be released in a public GitHub repository or linked archival repository upon acceptance, subject to dataset redistribution constraints, to facilitate reproducibility.

## Supplementary Materials

The following supporting information can be downloaded at: https://www.mdpi.com/article/10.3390/brainsci16070723/s1. The supplementary package includes per-seed results, fixed seeds, split protocols, split and control-pairing indices, training configuration summaries, statistical test files, training-log summaries, dataset/IP statement, and core source-code files.
